# Supplementary figures and images for: Immunological and prognostic significance of novel ferroptosis-related genes in soft tissue sarcoma
Source: PLoS One. 2022 Jan 4;17(1):e0262234. doi: 10.1371/journal.pone.0262234 (PMC8726495; doi:10.1371/journal.pone.0262234)

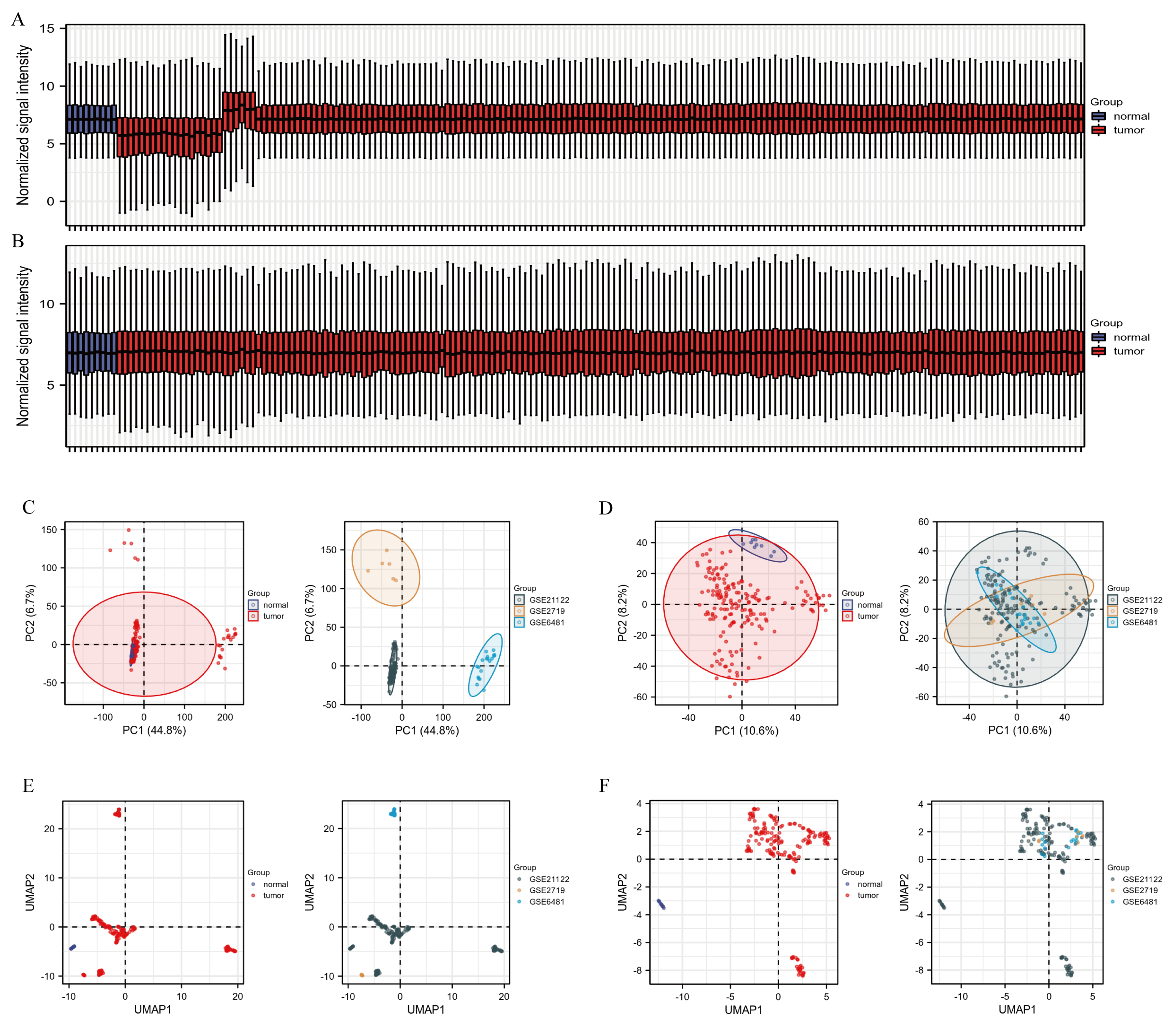

Supplement: S1 Fig — Comparison of box plots (A-B), PCA plots (C-D) and UMAP plots (E-F) before and after data pre-processing. (TIF) [file pone.0262234.s001.tif]
