# Supplementary material for: Immunological and prognostic significance of novel ferroptosis-related genes in soft tissue sarcoma
Source: PLoS One. 2022 Jan 4;17(1):e0262234. doi: 10.1371/journal.pone.0262234 (PMC8726495; doi:10.1371/journal.pone.0262234)
Supplement: Attachment — Submitted filename: Response to Reviewers.docx [file pone.0262234.s002.docx]

Dear editor,

Thank you very much for your letter and advice. We have revised the paper, and would like to re-submit it for your consideration. We have addressed the comments raised by the reviewers, and the amendments are highlighted in red in Revised Manuscript with Track Changes.

We hope that the revision is acceptable, and we look forward to hearing from you soon.

With best wishes,

Yours sincerely,

We would like to express our sincere gratitude to the reviewers for their constructive and positive comments. It is an honor for our manuscript to receive such rigorous and meticulous guidance. Explain accordingly one by one:

**Reviewer #1 comments:**

-Authors should clearly specify which samples they compared to identify genes differentially express in STS. Did they compare tumor vs normal tissues? Thank you for your insightful suggestion. We have added a detailed explanation and red-flagged it in the method section of the article.

-Authors should add in the main text a table reporting the name of the 40 ferroptosis-related genes along with their fold change. This is a great recommendation. We have added Table 1 to show that 40 ferroptosis-related genes.

-Results reported in figure 4B are not commented in the text. Authors should discuss the differential expression of the 6 hub genes between male and female, leiomyosarcoma and other STS subtypes, different disease progression. Thanks for the comment. We have added a detailed explanation of the clinical relevance of the hub gene in the results section of the article and have highlighted it in red.

-Authors should comment and discuss gene sets differentially enriched in STS TCGA samples divided on the basis of expression of the 6 hub genes. Thanks for your reminder. We have added a description of the GSEA results in the results section of the article and discussed the relevant results in the discussion section. All of the above changes are highlighted in red in the article.

-Paragraphs entitled ‘Association of hub genes expression and ICGs’ and ‘Association of hub genes expression and immune cells infiltration’ should be moved at the end of Results section. Thank you for your suggestion. They have been moved at the end of Results section.

-Authors should specify which deconvolution tool they used to estimate population of immune cells infiltrating the tumors. Thanks for your reminder. We have added the relevant information and red-flagged it in the method section of the article.

-‘Immunohistochemical validation’ paragraph should be removed, as in my opinion it does not represent a validation of results obtained by in silico analysis. Thanks for the comment. ’Immunohistochemical validation’ paragraph has been removed.

-Extensive editing of English language is required. Thank you for your insightful suggestion. In response to the language issues in the article, we have carefully reviewed and gone through extensive revisions. Subsequently, we found multiple English language professionals to review and correct the grammar and vocabulary in our article. Thank you again for your reminder.

-In Figure 2, names of GO and KEGG networks are unreadable. May be authors should add them in the bar graph. Thank you for your suggestion. Since the names of both KEGG and GO analysis results are too long, and the size of Figure is limited to the extent that they cannot be shown clearly in the bar chart. Therefore, we chose to provide additional descriptions of the names and numbers of the KEGG and GO analysis results in the Results section of the article, which are highlighted in red.

-In Figure 3, color legend is missing. Please, add it. Thanks for the comment. We have added them in the Figure 3.

**Reviewer #2 comments:**

Thank you for putting together this comprehensive manuscript that identifies several novel targets for immunotherapy agents derived from the ferroptosis pathway in sarcoma. It will be interesting to how you intend to translate these findings into clinical trials. Thanks for your reminder. This is one of the few comprehensive analyses for soft tissue sarcoma (STS) with large samples, multiple subtypes and multiple databases. Ferroptosis, immune checkpoint, immune cell and prognostic model were integrated. Initially, we identified novel ferroptosis-related genes with prognostic value in STS. Furthermore, we searched out potential key immune checkpoints and immune cells, and revealed for the first time their possible immune relevance. Ultimately, we constructed two efficient predictive models for prognosis of STS patients.

The results of our current study are a guide to translate and carry out subsequent experiments. The first is to carry out relevant experiments around ferroptosis, and to carry out ROS, GPX4, and Fe level measurements in STS samples around the six ferroptosis-related hub genes we identified, and to verify the relationship with related pathway molecules based on the results of enrichment analyses to find upstream and downstream mechanisms. Second, subsequent experiments can be centered on precise immunotherapy of STS. Since the original view was that immunotherapy for STS was almost useless, immunotherapy for STS is now possible with the discovery of novel RCD mechanisms such as ferroptosis. Due to the heterogeneity of STS, immunotherapy around STS requires a very high degree of precision. It is crucial to clarify the subtypes of STS that are sensitive to immunotherapy and to specify the immune cells, immune checkpoint genes, and reciprocal genes involved in STS immunotherapy. The immune cells and immune checkpoint genes identified in our current study with the broad category of STS are theoretically applicable to immunological studies of all STS subtypes. In addition, we found the immunogenicity of HELLS for the first time, and HELLS plays an important role in immunological studies of STS as a ferroptosis-related gene, which also has an innovative guiding effect on the development of related experiments. The final aspect involved is the validation of the clinical data, and the 2 prognostic models we designed were applicable to cohort studies of all STS subtypes. Of course to the extent that the bioinformatics analysis we performed only served as a guide, the definitive conclusions need to be validated by a large number of follow-up experiments.
