# Supplementary material for: Immunological and prognostic significance of novel ferroptosis-related genes in soft tissue sarcoma
Source: PLoS One. 2022 Jan 4;17(1):e0262234. doi: 10.1371/journal.pone.0262234 (PMC8726495; doi:10.1371/journal.pone.0262234)
Supplement: Attachment — Submitted filename: Response to Reviewers v2.docx [file pone.0262234.s003.docx]

Dear editor,

Thank you very much for your letter and advice. We have revised the paper, and would like to re-submit it for your consideration. We have addressed the comments raised by the reviewers, and the amendments are highlighted in red in Revised Manuscript with Track Changes.

We hope that the revision is acceptable, and we look forward to hearing from you soon.

With best wishes,

Yours sincerely,

**Editor Comments:**

Please review your reference list to ensure that it is complete and correct. Thanks for the comment. We have checked the reference list and made sure it is complete and correct.

Regarding the generation of prognostic nomograms, some clarifications and modifications are needed. Thank you for the reminder. Your suggestion is very meaningful. We have conducted a self-review based on the checklist according to your guidance. We have added and highlighted the missing descriptions in the manuscript, such as the patient population that generated the nomogram. We found that we met most of the requirements in the checklist, except for our study which may lack further validation. On the one hand, our study is one of the few that covers almost all common subtypes of soft tissue sarcoma and is therefore highly universal; however, on the contrary, validation of such a wide range of subtype models may not be so easy to achieve. In our study, we performed C-indexes and Calibration curves validation. Of course we clearly recognize that this is not sufficient and we have added a description of the shortcomings of the constructed nomogram in the discussion section, but we believe that our nomogram may be of some value as a guide and reference for subsequent related studies. Also, we fully understand your suggestion to remove the nomogram part, and we will comply with your suggestion to remove this section if you still feel that it is not necessary to keep it.

**Reviewer #3 Comments:**

We would like to express our sincere gratitude to the reviewer for the constructive and positive comments. It is an honor for our manuscript to receive such rigorous and meticulous guidance. Explain accordingly one by one:

1. Authors should specify which metric they used for ranking genes in GSEA. Thank you for your insightful suggestion. We have added a description of the GSEA ranking methodology in the results section.
2. They should make explicit in supplementary figure B and C which samples belonged to the same dataset. Thank you for your valuable comments. We have considered what you said before, but because the sample size included in our study was too large, adding 2 legends to one figure would have made the images very cluttered. After much testing and comparison, we finally chose the optimal approach and recreated the Figure S1. We divided the original image into 2 images and used different legend for each image.
3. Authors should maintain the same color scheme. Thanks for your reminder. We recreated the Figure 1 to ensure that the legend colors of the positive and negative expressions of the volcano plot, heat map, venn diagram, and survival curves remain consistent. We also clarified the description of the Y-label of the volcano plot. Also we added the missing legend to the heat map.
