# Supplementary material for: Immunological and prognostic significance of novel ferroptosis-related genes in soft tissue sarcoma
Source: PLoS One. 2022 Jan 4;17(1):e0262234. doi: 10.1371/journal.pone.0262234 (PMC8726495; doi:10.1371/journal.pone.0262234)
Supplement: Attachment — Submitted filename: Response to Reviewers v3.docx [file pone.0262234.s004.docx]

Dear editor,

Thank you very much for your letter and advice. We have revised the paper, and would like to re-submit it for your consideration. We have addressed the comments and the amendments are highlighted in red in Revised Manuscript with Track Changes.

We hope that the revision is acceptable, and we look forward to hearing from you soon.

With best wishes,

Yours sincerely,

**Editor Comments:**

- Please review your reference list to ensure that it is complete and correct.

Thanks for the comment. We have checked the reference list and made sure it is complete and correct.

**Additional Editor Comments:**

- The statement in the conclusion of abstract and manuscript about nomogram should be removed.

Thank you for your suggestion. We have removed the statement of nomogram in the conclusion section.

- Can you specify whether nomograms were generated on primary or metastatic or both tumours and patients? This should be clearly stated in the results section and discussion.

Thank you for your reminder. We have clarified this issue in the result section and in the discussion section.
